# Land Use and Climate Change Accelerate the Loss of Habitat and Ecological Corridor to Reeves's Pheasant ( *Syrmaticus reevesii* ) in China

**DOI:** 10.1002/ece3.70618

**Published:** 2024-11-26

**Authors:** Qingqing He, Shan Tian, Junqin Hua, Zhengxiao Liu, Yating Liu, Ting Jin, Jiliang Xu

**Affiliations:** ^1^ State Key Laboratory of Efficient Production of Forest Resources Beijing Forestry University Beijing China; ^2^ School of Ecology and Nature Conservation Beijing Forestry University Beijing China; ^3^ China Natural‐Harmony Blueprint Technology Co. Ltd. Guangzhou China

**Keywords:** conservation prioritization, ecological corridor, habitat, Reeves's pheasant

## Abstract

Human activity and climate change are widely considered to be primarily responsible for the extinction of Galliformes birds. Due to a decline in population, the Reeves's pheasant ( *Syrmaticus reevesii* ), a member of the Galliformes family, was recently elevated to first‐class national protected status in China. However, determining the causal factors of their extinction and carrying out protection measures appear to be challenging owing to a lack of long‐term data with high spatial and temporal resolutions. Here, based on a national field survey, we used habitat suitability models and integrated data on geographical environment, road development, land use, and climate change to predict the potential changes in the distribution and connectivity of the habitat of Reeves's pheasant from 1995 to 2050. Furthermore, ecological corridors were identified using the minimum cumulative resistance (MCR) model. The prioritized areas for habitat restoration were determined by integrating the importance indices of ecological sources and corridors. Our results indicated that both land use and climate change were linked to the increased habitat loss for the Reeves's pheasant. In more recent decades, road construction and land use changes have been linked to a rise in habitat loss, and future climate change has been predicted to cause the habitat to become even more fragmented and lose 89.58% of its total area. The ecological corridor for Reeves's pheasant will continue to decline by 88.55%. To counteract the negative effects of human activity and climate change on the survivorship of Reeves's pheasant, we recommend taking immediate actions, including bolstering cooperation among provincial governments, restoring habitats, and creating ecological corridors among important habitats.

## Abstract 2

Study results indicate that both intensified land use and climate change were associated with the increased habitat loss of the Reeves's pheasant. In more recent decades, human activities have been linked to a rise in local extinction, and future climate change is predicted to cause the habitat and ecological corridor to lose close to 89.5%.

## 1 Introduction

Global biodiversity has experienced a rapid decline since the onset of modern times (Rahbek and Colwell 2011), which poses significant threats to natural ecosystems and the conservation of biodiversity (Hooper et al. 2012). China is recognized as one of the countries with the most diverse avian populations, hosting a total of 1445 bird species, of which a significant number are endemic (Zheng 2017). However, many bird habitats have been fragmented or lost as a result of climate change and alterations in land use (Ubachs 2016). Currently, there are 394 bird species that are classified as endangered in China (Huang et al. 2021). Land use and climate change are considered to be two primary factors causing habitat fragmentation and local extinctions of animals (Dirzo et al. 2014). Reduced habitat connectivity may result in a decrease in effective population size, the occurrence of genetic bottlenecks, inbreeding depression, and potential population extinctions (Fagan 2002; Benson et al. 2016). Various strategies have been implemented for habitat conservation and restoration, including initiatives designed to protect forest ecosystems through the establishment of protected areas, the development of effective ecological corridors to reduce habitat fragmentation, and efforts to enhance genetic diversity. These measures are essential for preventing avian population declines and habitat loss (Colyn et al. 2020; Liu, Huang, and Tang 2021; Chen, Li, and Liu 2022). Nevertheless, only limited financial resources are allocated for biodiversity conservation. Therefore, prioritizing critical patches that serve as connectivity providers can enhance the overall effectiveness of biodiversity protection across fragmented landscapes (Visconti et al. 2010; Gurrutxaga and Saura 2014). The challenges related to the protection and maintenance of biodiversity are inherently dynamic. Therefore, it is necessary to continuously reassess and project the state of habitat distribution and connectivity to ensure that these habitats can effectively respond to the impacts of climate change and land use alterations (Li et al. 2024).

Different patches typically serve distinct functions in the maintenance of overall connectivity, which is attributable to their specific characteristics. Generally, only a limited number of patches have significantly contributed to sustaining habitat connectivity (Han et al. 2022). The integration of the importance of habitat patches and the capacity of individuals to access appropriate habitats, along with the establishment of ecological corridors connecting these habitats, is a critical element in the restoration of habitat connectivity, which may significantly influence the persistence of populations within fragmented landscapes (Colyn et al. 2020; Han et al. 2022; Gao et al. 2023). At present, three main approaches to identify ecological corridors are as follows: the graph‐based network approach (Minor and Urban 2008); the minimum cumulative resistance (MCR) model (Peng et al. 2019; Liu, Huang, and Tang 2021); and circuit theory (Peng, Zhao, and Liu 2017). However, current approaches to constructing ecological corridors, which consider protected areas or forests as ecological source areas, do not inherently account for the habitat needs of each species (Peng et al. 2019). In addition, they only treat land use as ecological resistance, leading to simplified resistance surfaces (Peng et al. 2019).

The Galliformes are among the most threatened groups of birds due to direct exploitation for food, cultural practices, and habitat loss (Keane, Brooke, and McGowan 2005). According to Grainger et al. (2018), 27% of species in this group are globally threatened. Reeves's pheasant ( *Syrmaticus reevesii* ) belongs to the Galliformes order and is a flagship species for conservation initiatives in certain mountain ranges in Central China where it was previously abundant (Tian, Xu, and Wang 2020). Due to the growing impact of human activities and climate change, the habitat of Reeves's pheasant has become increasingly fragmented (Feng et al. 2015). The previously continuous population of Reeves's pheasant has now been fragmented into two isolated geographic subpopulations, which are also patchy and scattered (Zheng 2015). Due to habitat loss and rapid population declines, the species was previously listed as “Vulnerable” on the IUCN Red List (IUCN 2020) and as a first‐class protected animal in China (National Forestry and Grassland Administration 2021). In order to address the issue of habitat fragmentation for Reeves's pheasant, the construction of ecological corridors has been proposed (Han et al. 2022; Lu et al. 2023). However, the prioritized area for corridor construction has not been determined.

The restoration of habitat should prioritize historical records and existing locations to ensure that both short‐term and long‐term changes in the availability of suitable habitat do not decrease in response to current and future human activities and climate change (Li et al. 2018; Banks‐Leite et al. 2020). So the alterations in habitat and ecological corridors for Reeves's pheasant in response to habitat changes require immediate attention. The objectives of this study were to: (1) assess habitat changes for the Reeves's pheasant in 1995, 2020, and 2050 under different climatic and land use conditions; (2) identify ecological connectivity for the Reeves's pheasant in 1995, 2020, and 2050; and (3) screen important areas for the restoration of ecological connectivity for the Reeves's pheasant.

## 2 Method

### 2.1 Study Area and Species Occurrence Data Collection

The primary objective of this study was to analyze changes in habitat connectivity and ecological corridors for the Reeves's pheasant covered in all areas of China in 1995, 2020, and projected for 2050. The occurrence data for the Reeves's pheasant utilized in this study were collected from both field surveys and the Global Biodiversity Information Facility (GBIF). The field survey data were obtained from the survey on Reeves's pheasant conducted during the period of 2018 to 2019 in China by us Tian et al. (2022). The survey area encompassed 49 counties involving eight provinces across China, and a total of 219 line transects were established, with lengths ranging from 850 to 3600 m, all of which were surveyed on foot in the field. A fixed width of 50 m on each side of the line transects was surveyed to assess occurrence by direct sightings and indirect presence evidence (e.g., feathers, nest sites, and wing‐whirring sounds) of Reeves's pheasant. The detailed description of the field survey methodology and investigated counties were detailed in the previous study (Tian et al. 2022). The occurrence locations within 1 km were excluded to avoid pseudoreplication and spatial autocorrelation using the R package “geosphere” in R v4.3.1 (R Core team 2022), as the average maximum home range of Reeves's pheasant measures 1.05 km^2^ (Zhou et al. 2017; Tian, Xu, and Wang 2020). A total of 171 field survey occurrence locations were retained.

It is difficult to identify historical habitat distributions in the assessment of species habitats due to a lack of data (Murphy and Smith 2021). This inadequacy significantly impedes the understanding of habitat distribution changes. The GBIF serves as a repository for information regarding the locations and temporal records of species, encompassing a range of data from museum specimens collected in the 19th century to more recent observations (Liu et al. 2023). Therefore, we acquired occurrence data for Reeves's pheasant in China from the 19th century through the GBIF (https://www.gbif.org/). The downloaded occurrence data were meticulously proofread against published literature and verified by experts to ascertain whether the reported sampling sites corresponded to the actual locations where the data were collected (Zhou, Xu, and Zhang 2015). As previously mentioned, we excluded data for distributions within a 1 km radius, resulting in the acquisition of 25 occurrences of Reeves's pheasant from the GBIF. Furthermore, a total of 171 occurrence locations obtained from the field survey were integrated to ensure comprehensive coverage of the distribution of Reeves's pheasant in 1950. Thus, 196 occurrence locations were retained for modeling habitat suitability.

### 2.2 Environmental Variables

We first acquired bioclimatic variables for the Years 1995 and 2020 from the WorldClim database (http://www.worldclim.org/), including temperature and precipitation data for each of the 12 months for both years, with a spatial resolution of 30 s. Utilizing the “biovars” function from the R package “dismo” (Hijmans et al. 2023) in R v4.3.2 (R Core Team, 2023), we computed several key climate variables: mean annual temperature (Bio 1), mean temperature of the warmest quarter (Bio 10), mean temperature of the coldest quarter (Bio 11), mean annual precipitation (Bio 12), precipitation of the driest quarter (Bio 16), and precipitation of the wettest quarter (Bio 17) for both 1995 and 2020. These variables were selected due to their established importance in delineating climate space for various species and were determined to be nonhighly correlated factors (Elsen et al. 2020; Asamoah, Beaumont, and Maina 2021). Additionally, land use data for 1995 and 2020 were acquired from the European Space Agency (http://maps.elie.ucl.ac.be/CCI/viewer/), with a spatial resolution of 300 m. Furthermore, we utilized road data in 1995 and 2020 obtained from the Resource and Environment Science Data Centre of the Chinese Academy of Sciences (https://www.resdc.cn/).

The future bioclimatic variables encompass four scenarios: SSP 1–2.6, SSP 2–4.5, SSP 3–7.0, and SSP 5–8.5. Among these, SSP 2–4.5 is considered the most likely scenario to be realized under the current conditions of enforced emission reduction policies (Thompson et al. 2011). Consequently, the bioclimatic variables Bio 1, Bio 10, Bio 11, Bio 12, Bio 16, and Bio 17 for the 2050 scenarios of SSP 2–4.5 were downloaded from WorldClim with a spatial resolution of 30 s for subsequent modeling analyses. The land use data in 2050 were obtained from the global land projection based on the Plant Functional Types dataset (Global PFT‐based land projection) under the SSPs‐RCPs framework, which has a spatial resolution of 1 km. This dataset is consistent with the latest IPCC‐coupled socioeconomic and climate change scenarios (SSP‐RCP) (Chen, Li, and Liu 2022). Furthermore, the PFT‐based dataset offers a more comprehensive array of land‐type information, and its classification criteria closely resemble those of the land use data in 1995 and 2020 (Chen, Li, and Liu 2022). We opted to download land use data corresponding to the SSP 2–4.5 scenarios, as they align with the future climate scenarios. Given that the road distribution data in 2050 are currently unavailable, we used the road data in 2020 instead to minimize potential errors.

Additionally, we employed the digital altitude model obtained from the Chinese Academy of Sciences Resource Environmental Data Center (https://www.resdc.cn), which possesses a spatial resolution of 300 m. Slope and aspect were derived from the digital elevation model using ArcGIS 10.7. Utilizing data at a resolution that approximates the dispersal capacity of organisms for habitat assessment significantly enhances the validity of the model (Han et al. 2022). The 300‐m‐resolution data are aligned with the average distance of 500 m observed in the daily movements of Reeves's pheasant. Therefore, this study employed the resampling tool in ArcGIS 10.7 to standardize all environmental raster data to a resolution of 300 × 300 m. The variance inflation factor (VIF) was utilized to address the collinearity of environmental variables, and all variables with VIF values less than 10 were retained for subsequent analysis (Liu et al. 2023). The environmental dataset included: Bio 1, Bio 10, Bio 11, Bio 12, Bio 16, Bio 17, land use, road distance, altitude, slope, and aspect, which have been selected for modeling habitat suitability.

### 2.3 Habitat Identification

To reduce the uncertainty associated with predictions based on a single model and increase the effectiveness of conservation efforts, we adopted the ensemble modeling approach based on multimodel predictions (Jones‐Farrand et al. 2011) for the occurrence and suitable habitat. We used the “dismo” (Hijmans et al. 2023) package for species distribution modeling (SDM) in R v4.3.2. Three modeling algorithms, including additive models (GAMs), and two machine learning methods (random forest [RF] and maximum entropy [MaxEnt]) were selected because they have been reported to exhibit high performance in species distribution assessments (Razgour et al. 2019; Hu et al. 2022).

To identify habitat for Reeves's pheasant, this study utilized 75% of the records from the occurrence dataset as the training set and 25% as the test set. The Jackknife method was employed to quantify the importance of each environmental factor (Coetzee et al. 2009) in relation to the habitat of Reeves's pheasant. Then, we used true skill statistics (TSS) (Allouche, Tsoar, and Kadmon 2006) and the values of the area under a receiver operating characteristic curve (AUC) (Elith et al. 2006) to calibrate and validate the robustness of the evaluation using the three models (Tian et al. 2022). The AUC and TSS values varied between 0.5 and 1. Models exhibiting AUC values below 0.8 and TSS below 0.7 were considered “poor” performance (Zhang et al. 2018; Mi et al. 2023), and were excluded from the final model predictions. We computed the weights for the predictions generated by each model according to its AUC score. This was achieved by subtracting 0.5, which represents the random expectation, and subsequently squaring the resulting value. This methodology afforded greater weight to models exhibiting higher AUC values (Tian et al. 2022).

### 2.4 Quantifying Changes in Habitat Connectivity

Habitat connectivity is a major concern for the survival of wildlife populations and the risk of extinction (Kramer‐Schaadt et al. 2004). The integral index of connectivity (IIC) and probability of connectivity (PC) were calculated based on the estimated dispersal distance (Equations [1] and [2] are used to evaluate the habitat connectivity between two randomly selected patches from the entire fragmented landscape) (Pascual‐Hortal and Saura 2006; Saura and Pascual‐Hortal 2007).

(1) $$\mathrm{IIC}=\frac{\sum_{i=1}^{n}\sum_{j=1}^{n}a_{i}a_{j}/\left(1+{\mathrm{nl}}_{\mathrm{ij}}\right)}{A_{L}^{2}},0<\mathrm{IIC}<1$$

(2) $$\mathrm{PC}=\frac{\sum_{i=1}^{n}\sum_{j=1}^{n}a_{i}\times a_{j}\times P_{\mathrm{ij}}/\left(1+{\mathrm{nl}}_{\mathrm{ij}}\right)}{A_{L}^{2}},0<\mathrm{PC}<1$$

where *n* is the total number of ecological patches; *a*_*i*_ and *a*_*j*_ are the areas of patches *i* and *j*; *nl*_*ij*_ denotes the number of links in the shortest path (topological distance) between patches *i* and *j*; *P*_*ij*_ is the maximum product probability of all paths between patches *i* and *j*; and *A*_*L*_ is the total landscape area. Two dispersal distances, specifically 500 and 1000 m, were selected to calculate IIC and PC values as they represent the average and maximum movement distances recorded for Reeves's pheasant over the survey of a week (Tian et al. 2022).

### 2.5 Ecological Corridor Identification

#### 2.5.1 Ecological Source Identification

In this study, we used the morphological spatial pattern analysis (MSPA) segmentation method, which is integrated into the Guidos Tool Box (Vogt and Riitters 2017) developed by the European Commission Joint Research Centre (JRC), to identify core areas in the habitat raster. The MSPA classification routine begins by identifying core areas, using user‐defined rules to determine connectivity and edge width. Habitats will be classified by MSPA into eight distinct types: core, islet, loop, bridge, perforation, missing, edge, and branch, based on edge width. The core area serves as a substantial habitat for species, which is crucial for biodiversity conservation and represents a potential source for the establishment of ecological networks (Chen et al. 2024). Studies have shown that a green space area threshold of 35 km^2^ is important in supporting the survival of Reeves's pheasant population (Tian et al. 2022). The index was used to exclude core area patches smaller than 35 km^2^ and to identify the remaining core areas as ecological sources.

#### 2.5.2 Ecological Resistance Surface Construction

The resistance surface reflects the degree of resistance and the heterogeneity of the landscape that influence species movement. In this study, habitat quality was transformed into a resistance surface utilizing the Raster Calculator in ArcGIS 10.7 (Cushman et al. 2013). The habitat quality data were derived from Section 2.3, titled “Habitat identification.” This approach also prevents the resistance surface from being in binary states and avoids simplification of resistance surfaces due to consideration of land use types only (Gao et al. 2023).

#### 2.5.3 Construction of Corridor Systems Under Different Scenarios

After identifying the source sites and resistance values using the SDM, we established ecological corridors between the core patches based on the MCR model (McRae et al. 2012) and circuit theory (McRae et al. 2008). We employed linkage mapper (LM) to identify ecological corridors. LM is specifically designed to facilitate regional analyses of wildlife habitat connectivity. It can be utilized to delineate ecological corridors between ecological source sites by employing the MCR and circuit theory (Peng et al. 2019). The limiting threshold of cost‐weighted value was set by default at 20,000 to effectively account for corridors connecting individual ecological source patches (Colyn et al. 2020).

### 2.6 Extraction of Important Ecological Sources and Ecological Corridor

In this study, we identified important ecological sources that significantly influenced overall landscape connectivity. The significance of individual patches (dPC) (Equations [3]) in maintaining overall connectivity was assessed in accordance with the methodology proposed by Saura and Pascual‐Hortal (2007), utilizing following formula:

(3) $$\mathrm{dPC}=\frac{\mathrm{PC}-{\mathrm{PC}}^{\prime }}{\mathrm{PC}}\times 100\%,0<\mathrm{dPC}<1$$

where *PC* and ${\mathrm{PC}}^{\prime }$ represent the values of the “Probability of Connectivity” index when an individual patch is present (PC) and when it is removed (${\mathrm{PC}}^{\prime }$) from the studied landscape. The connectivity and important source of the Reeves's pheasant were determined using Conefor Sensinode 2.2 (Saura and Torné 2009).

**FIGURE 1:**
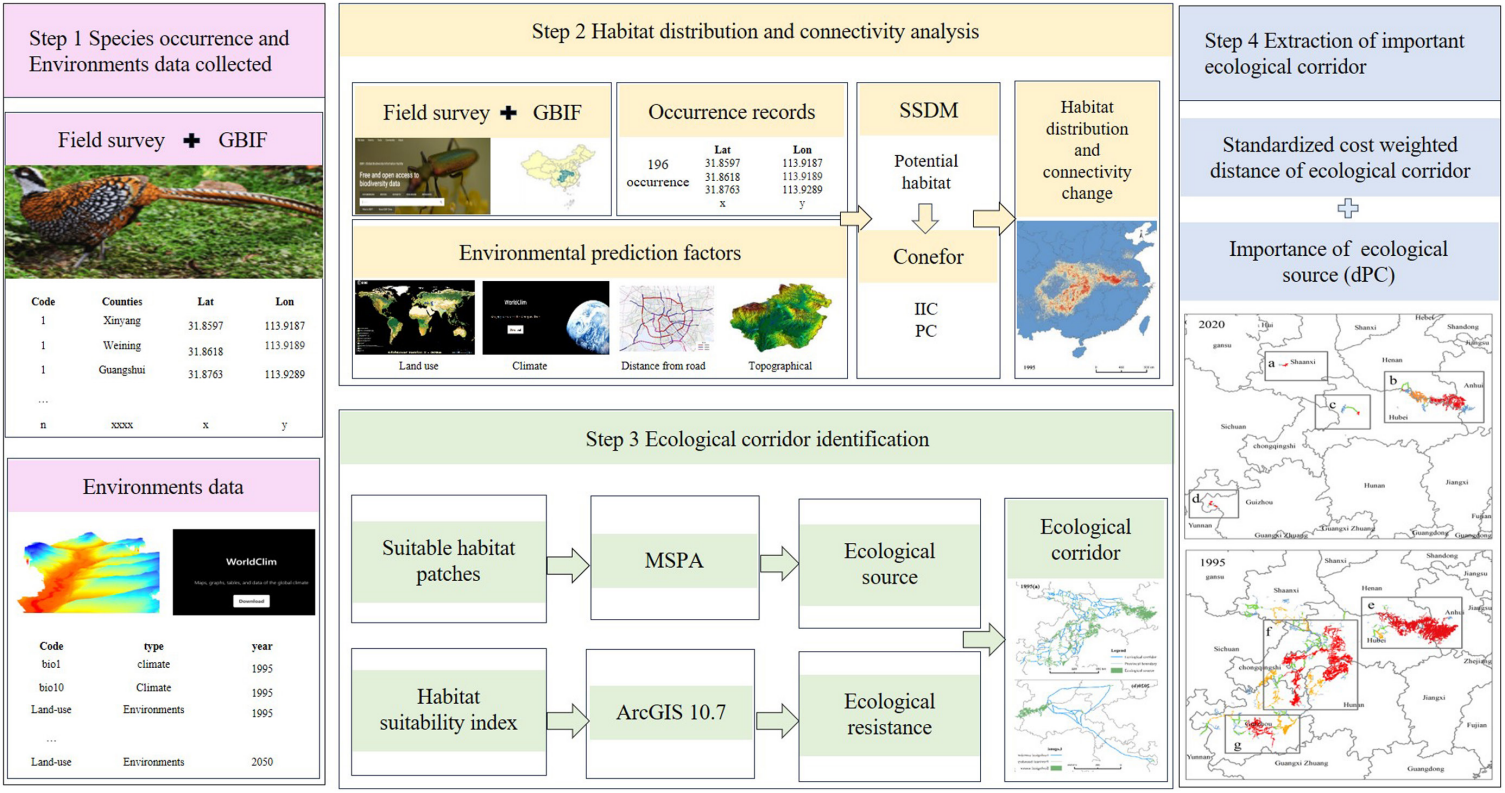
Research technology roadmap.

The significant corridors identified by the Linkage Pathways Tool in the LM function as edges in the topological network structure. The ecological corridor is weighted by the standardized cost‐weighted distance, indicating the varying strength of interactions between nodes and revealing the differing transmission capacities of ecological corridors within ecological networks (Gao et al. 2023). The dPC values of ecological sources and the cost‐weighted distances of corridors were subsequently input into ArcGIS 10.7 and classified into four levels of importance: 0%–25%, 25%–50%, 50%–75%, and greater than 75% (Han et al. 2022; Gao et al. 2023). The data analysis processes outlined above can be referenced in the accompanying Figure 1.

## 3 Results

### 3.1 Habitat Area and Connectivity Changes

**FIGURE 2:**
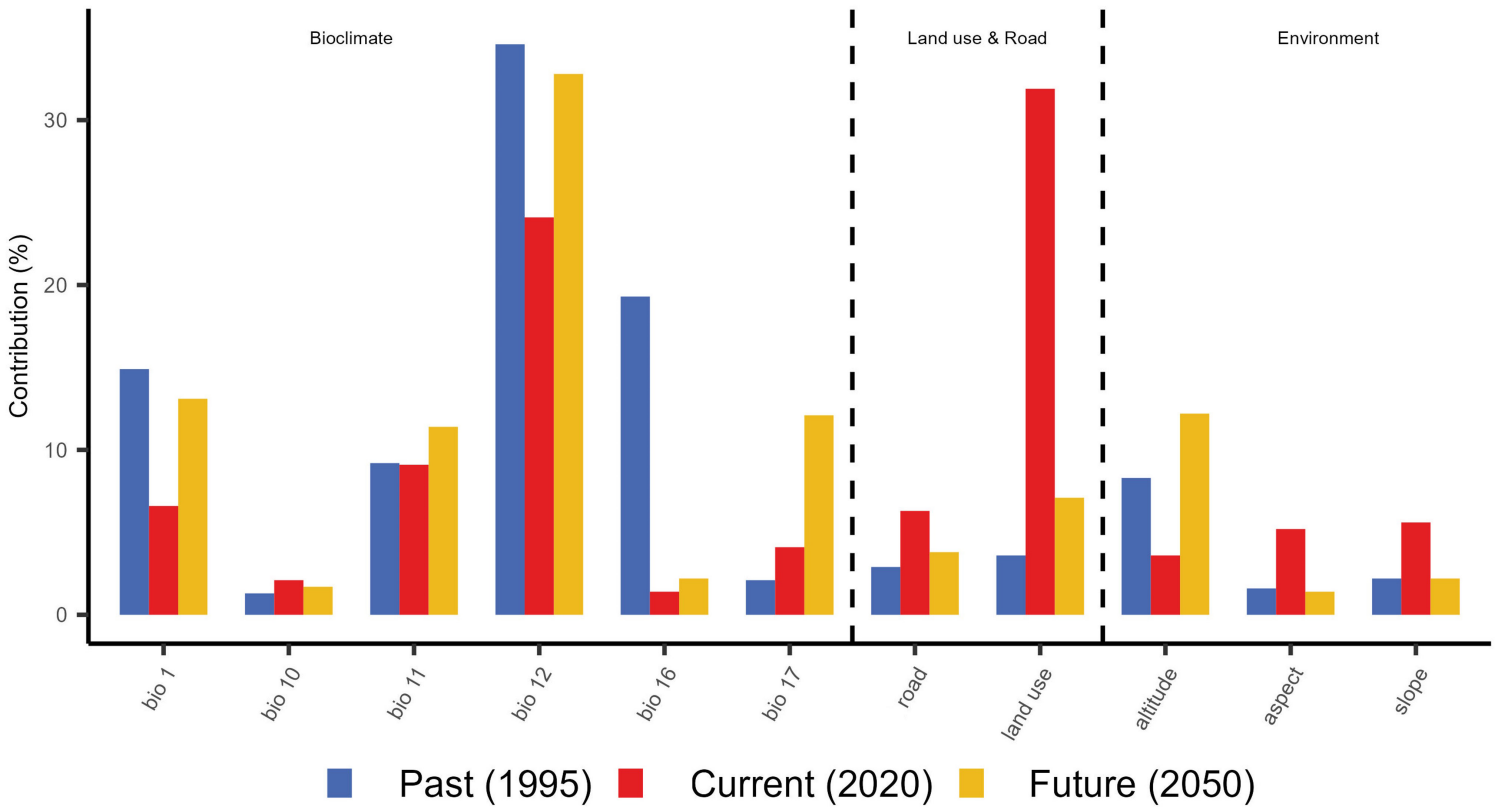
Percentage contribution of different variables to suitable habitat for Reeves's pheasant in the past (1995), present (2020), and future (2050). Bio 1: Mean annual temperature; Bio 10: Mean temperature of the warmest quarter, Bio 11: Mean temperature of the coldest quarter, Bio 12: Mean annual precipitation, Bio 16: Precipitation of the driest quarter, and Bio 17: Precipitation of the wettest quarter.

**FIGURE 3:**
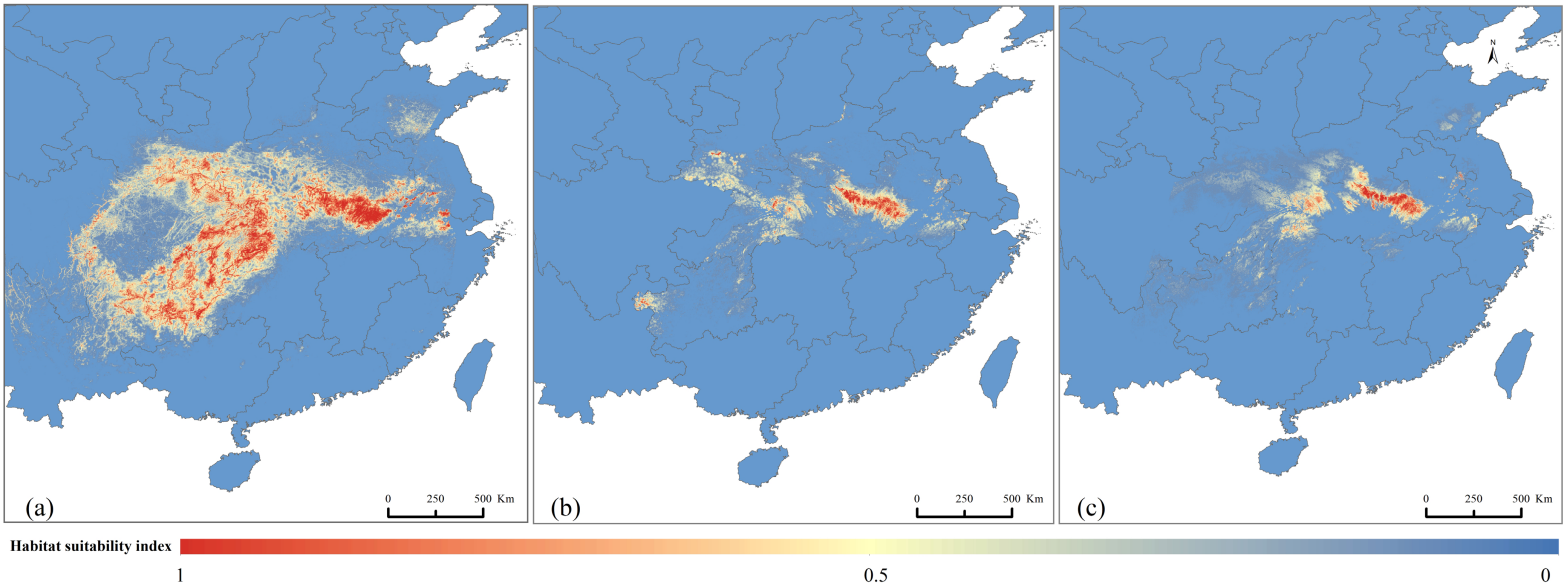
Habitat distribution of Reeves's pheasant in different years; (a–c) represent the habitat distribution of Reeves's pheasant in 1995, 2020, and 2050, respectively.

The ensemble model showed that RF and MaxEnt models had good explanatory power for habitat assessment in 1950 (AUC = 0.91, TSS = 0.86), 2020 (AUC = 0.95, TSS = 0.91), and 2050 (AUC = 0.87, TSS = 0.8). Land use contributed the most to Reeves's pheasant's habitat suitability in 2020, with a contribution of 31.9%. However, the climate factor Bio 12 had the greatest effect on Reeves's pheasant's habitat suitability in 1995 and 2050, with contributions of 34.6% and 32.5%, respectively (Figure 2). Due to the synergistic effect of climate and land use changes, the area of suitable habitat decreased by 89.58% from 1995 to 2050. It peaked at 91,571 km^2^ in 1995, and subsequently declined to 15,436 km^2^ in 2020, with a further projected decrease to 10,002 km^2^ in 2050 (Figure 3).

Both the PC and the IIC increased as dispersal distance increased from 500 to 1000 m. However, habitat connectivity for Reeves's pheasant declined progressively in 1995, 2020, and 2050, at the dispersal distance of 500 and 1000 m (Table 1), indicating an overall decrease in landscape connectivity of Reeves's pheasant's habitat from 1995 to 2050.

**TABLE 1:** Probability of connectivity index (PC) and integral index of connectivity (IIC) normalized for the two dispersal distances (500 and 1000 m) selected in this study.

Dispersal distance (m)	PC			IIC		
	1990	2020	2050	1990	2020	2050
500	91	21	23	90	27	31
1000	92	43	48	92	49	51

### 3.2 Results of Corridor Construction

**FIGURE 4:**
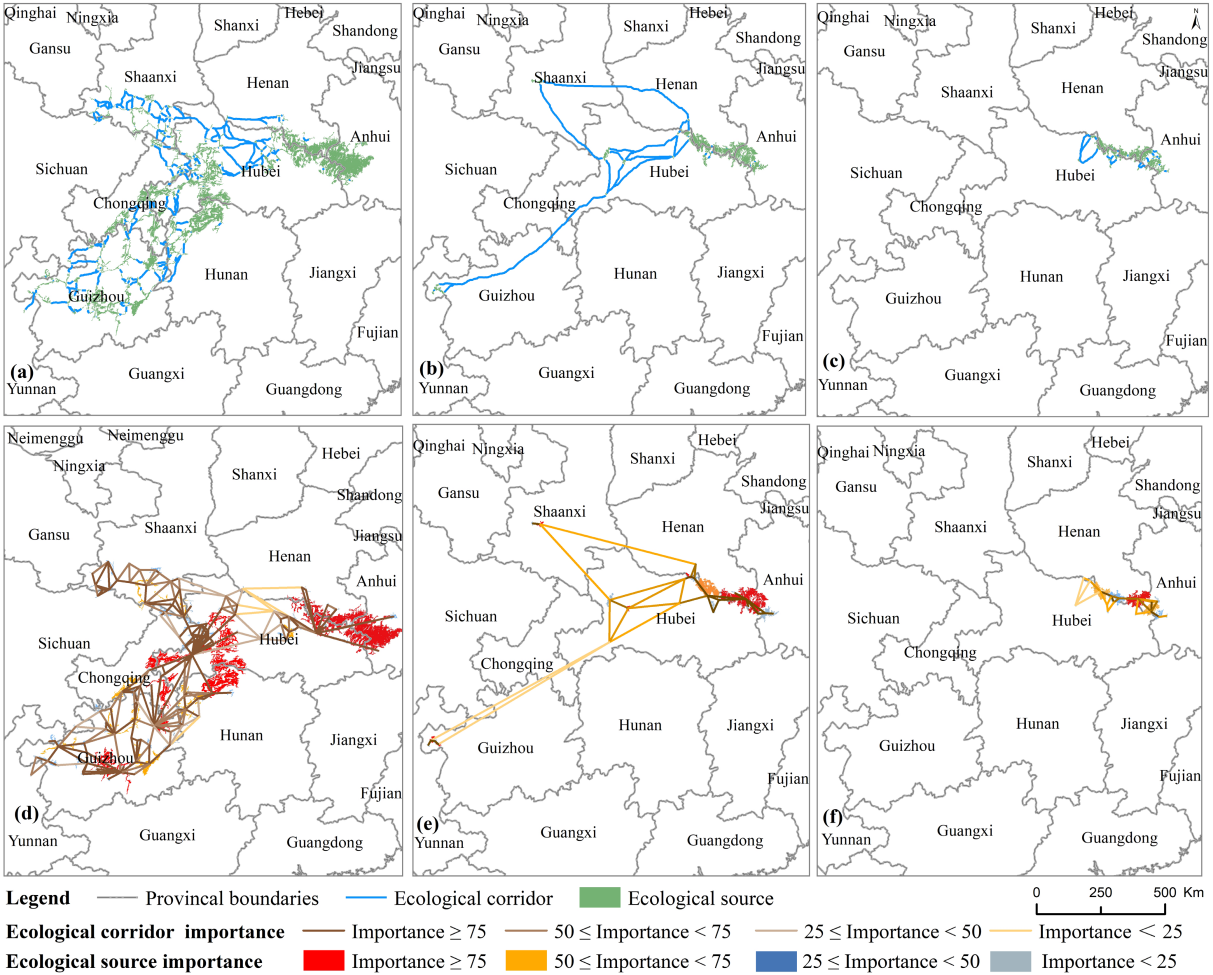
The distribution of ecological corridors and ecological sources for the Reeves's pheasant across various years, along with the classification of the significance of ecological sources and corridors; (a–c) represent the ecological corridors and ecological source distributions of Reeves's pheasant in 1995, 2020, and 2050; (d–f) represent the results of the ecological corridor and ecological source importance classification of the Reeves's pheasant in 1995, 2020, and 2050, respectively.

The results of MSPA showed that in 1995, there were a total of 121 ecological sources for Reeves's pheasant in China, covering a total area of about 72,831 km^2^ (Table S1). The 332 ecological corridors formed a complex network connecting the southwestern, northwestern, and central parts of the Reeves's pheasant's habitat in China. The ecological corridors had an average length of 5467 m (ranging from 9.82 to 61,289 m) (Figure 4a).

In 2020, there were a total of 21 ecological sources for the Reeves's pheasant in China, covering a combined area of approximately 13,239.80 km^2^ (Table S1), and 55 ecological corridors were identified with an average length of 41,615 m (ranging from 35 to 272,884 m). The ecological corridors were essentially divided into three isolated regions mainly located in Central China, with a small portion located in southwestern and northwestern China (Figure 4b).

There will be a total of 16 ecological sources for Reeves's pheasant in China in 2050, covering a combined area of approximately 8325.80 km^2^ (Table S1). In 2050, there will be only 38 ecological corridors in China, with an average length of 5263 m (ranging from 65 to 27,057 m) (Figure 4c). The ecological sources and corridors will be only located in the central part of China.

### 3.3 Extraction of Critical Ecological Sources and Important Ecological Corridors

**FIGURE 5:**
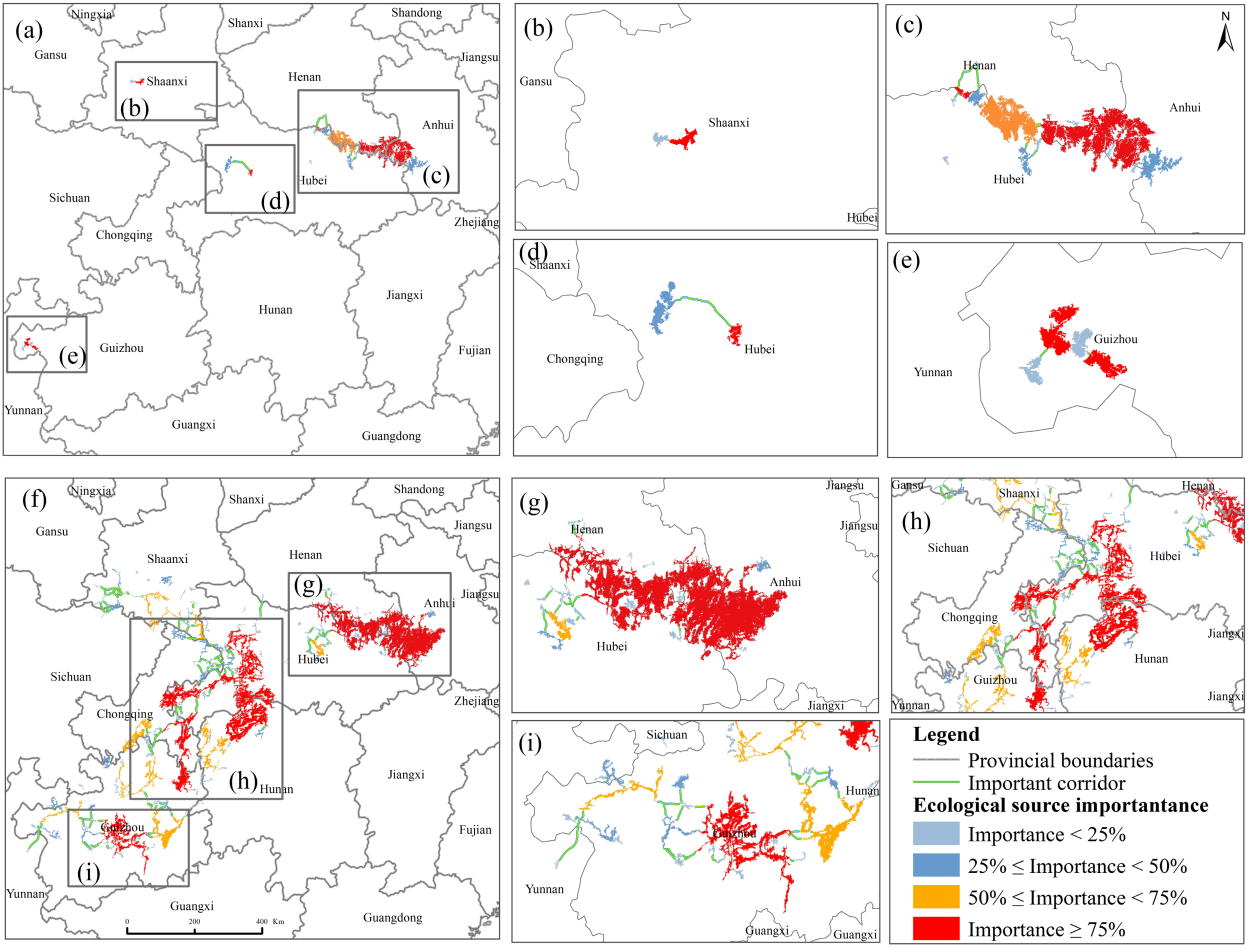
Distribution of important ecological corridors for Reeves's pheasant in 1995 and 2020; (a–e): Current (2020) areas of important habitat restoration and ecological corridor construction for Reeves's pheasant; (f–i): Historic (1995) Reeves's pheasant potential habitat restoration and ecological corridor construction area.

The dPC values suggested that the presence of five, four, and three habitat patches were the most important contributors to the habitat connectivity of Reeves's pheasant in the Years 1995, 2020, and 2050, respectively. Furthermore, an analysis of the cost‐weighted distance per corridor revealed the existence of 24, 18, and 8 important ecological corridors in 1995, 2020, and 2050, respectively (Figure 5).

This study analyzed critical habitat restoration areas and corridor construction areas for Reeves's pheasant in 1995 and 2020 (Figure 5). The important ecological sources and corridors for Reeves's pheasant in 2020 were predominantly situated in Guizhou, Hubei, Anhui, and Henan Provinces (Figure 5a–e). Historically, during the 1950s, northern Guizhou Province, northwestern Hunan Province, and northern Chongqing City also served as critical distribution areas of habitat and corridor (Figure 5f–i).

## 4 Discussion

### 4.1 Impacts of Land Use and Climate Change

It is widely known that the main direct drivers of biodiversity loss are habitat transformation (i.e., conversion to agricultural land), climate change, and overexploitation (e.g., hunting) (Banks‐Leite et al. 2020). More than 70% of the surviving forest is currently located less than 1 km from the edge of a nonforest ecosystem, according to previous studies that suggest both global warming and cooling can result in range shifts and local extinctions of animals (Li et al. 2018; Banks‐Leite et al. 2020). This study demonstrates that both land use and climate change are correlated with the significant habitat loss of the Reeves's pheasant from 1995 to 2050. Our study showed that the habitat of Reeves's pheasant experienced a substantial decline of approximately 83.14% from 1995 to 2020, with an anticipated further reduction of 89.58% by 2050.

During the past three decades, China has experienced a rapid increase in population, industrialization, and urbanization, as well as substantial land use changes at the local scale, thus imposing great pressure on animals (Wan et al. 2019). Significant land use changes such as cropland expansion and deforestation have not only resulted in the destruction of animal habitats but have also facilitated poaching activities. It is important to stress that the effective population size of Reeves's pheasant has declined by roughly 20% annually over the past few decades because of illegal hunting and habitat destruction (Zhou et al. 2017; Han et al. 2022). In addition, studies have shown that forest and wetland/water surface areas have declined significantly in China since 1995 (Sannigrahi et al. 2018). Since the habitat of Reeves's pheasant was more sensitive to land use changes than to climate change during 1995–2020, we conclude that land use changes and anthropogenic disturbances may have contributed to the rapid decline in Reeves's pheasant's habitat connectivity, in recent decades (Figure 3).

While the Reeves's pheasant's habitat is more sensitive to climate change during 1995 and 2020–2050, previous research has indicated that during the cold phase, climate change was positively correlated with an increase in local extinctions of large mammals, such as elephants ( *Elephas maximus* ) and pandas ( *Ailuropoda melanoleuca* ). In contrast, in the contemporary period, human disturbances have been linked to a rise in local extinction rates (Wan et al. 2019). These results may suggest that Reeves's pheasant's survival depended on specific environmental factors such as altitude, slope, and other broad‐scale climatic factors (such as temperature and rainfall) in the past (Xu et al. 2007; Zhou et al. 2017; Pecchi et al. 2019). Reeves's pheasants, which are severely threatened by human disturbance, are primarily found in fragmented landscapes with little connectivity, affording little chance for gene flow between subpopulations (Tian, Xu, and Wang 2020; Lu et al. 2023). Despite efforts to mitigate land use and climate change, our results indicated that, in response to extreme weather events and rising temperatures, the habitat of Reeves's pheasant experienced a reduction in both area and connectivity, accompanied by an increase in fragmentation. Therefore, without effective measures to improve habitat connectivity, the Reeves's pheasant in severely fragmented landscapes will continue to be at high risk of extinction.

### 4.2 Implication for Conservation

In this study, we assessed the changes in ecological corridor and ecological source of Reeves's pheasant and estimated their importance to landscape connectivity. Our research indicates that a concerted national initiative aimed at restoring native habitats is needed to address the habitat loss crisis of the Reeves's pheasant in China. Additionally, it is imperative to construct both natural and artificial corridors to facilitate connectivity among isolated subpopulations of the species (Figure 4).

Ecological corridors composed of sources and corridors are considered a sustainable landscape pattern that serves as an effective spatial pathway to maintain regional ecological security and promote sustainable development (Tang et al. 2023). Ecological sources may serve as critical stepping stones to facilitate long‐distance migration among populations, thereby mitigating isolation between these populations (Han et al. 2022). Protecting important ecological sources can reduce migration risks across the landscape (Prevedello, Almeida‐Gomes, and Lindenmayer 2018; Le Roux et al. 2018), increase ecological connectivity, and allow species to colonize new suitable areas (Saura, Bodin, and Fortin 2014; Herrera et al. 2017). Prioritizing habitat restoration for species involves constructing ecological corridors within their existing or historically important source areas (Banks‐Leite et al. 2020). This study analyzed important habitats for Reeves's pheasant in 1995, 2020, and 2050, revealing that the habitats in Guizhou and Shanxi Province are critical and endangered ecological sources. The ecological sources in Henan and Anhui Provinces are critical and stable, providing a refuge for Reeves's pheasant habitat and ecological security under changing climate and land use conditions (Figure 5).

The number of ecological corridors for Reeves's pheasant decreased with habitat loss, and the length of ecological corridors has increased since 1995. This may result in a loss of core breeding and/or foraging habitats, while habitat fragmentation imposes an additional impact on genetic diversity and population integrity (Jones‐Farrand et al. 2011). However, the unweighted complex networks assume that all corridors are equally accessible, which is inconsistent with actual topological conditions (Gao et al. 2023). In this study, we assigned weights to edges based on the cost–weight distances derived from resistance surfaces on corridors. This was done to demonstrate the important impact of weight on the network and to reflect the difference between weighted and unweighted networks (Figure 5). Two and one important ecological corridors were identified in Guizhou and Shaanxi Provinces, respectively. To prevent fragmentation and manage the area under a standardized regime, it is suggested that the concentration of important ecological sources and corridors in Henan and Anhui should be integrated. The habitat conservation for Reeves's Pheasant, as well as the establishment of ecological corridors, necessitates the coordination and collaboration of provincial governments.

Importantly, previous studies reported that climate and land use changes would also reduce the suitable habitats of other Galliformes species (Liu et al. 2023). The findings in our study are thus also expected to have significant implications for the conservation of other forest‐dwelling and ground‐nesting Galliformes species.

### 4.3 Limitation of This Study

Our results provide insights into the reason for the habitat loss of Reeves's pheasant and have important conservation implications. However, due to the precision limitation of our historical data and environmental elements (e.g., uncertainty of historical records, biased recording efforts in space and time, and land use or climate resolutions), conclusions should be cautiously interpreted. In addition, this study proposes a model for constructing ecological corridors for Reeves's pheasant, which can be adopted to assess individual Galliformes birds. This approach allows for the differentiated modeling of each ecological corridor and the identification of important habitat conservation and construction areas. Nevertheless, we are unable to include information on the dispersal abilities and preferences of Reeves's pheasant among different habitats due to unavailability of information. Understanding ecological habits of species can help to enhance the accuracy of ecological networks (Xu et al. 2023). As many scholars have pointed out, the construction of ecological corridors depends on various factors such as environmental conditions, habitat heterogeneity, population density, economic development, and resource availability (Abrahms et al. 2021; Dai, Liu, and Luo 2021). Therefore, interdisciplinary research that combines geography, ecology, economics, and movement ecology is needed.

## Author Contributions

**Qingqing He:** conceptualization (lead), resources (lead). **Shan Tian:** conceptualization (equal), investigation (equal), resources (supporting). **Junqin Hua:** investigation (equal), supervision (equal). **Zhengxiao Liu:** data curation (equal), supervision (equal). **Yating Liu:** data curation (equal), supervision (equal). **Jiliang Xu:** conceptualization (equal), funding acquisition (equal), supervision (equal), writing – review and editing (equal).

## Conflicts of Interest

The authors declare no conflicts of interest.

## Supporting information


**Table S1.** Area of landscape type based on morphological spatial pattern analysis (MSPA) in 1995, 2020, and 2050.

## Data Availability

The data that support the findings of this study are available from the corresponding author, [author initials], upon reasonable request.

## References

[ece370618-bib-0001] Abrahms, B. , E. O. Aikens , J. B. Armstrong , W. W. Deacy , M. J. Kauffman , and J. A. Merkle . 2021. “Emerging Perspectives on Resource Tracking and Animal Movement Ecology.” Trends in Ecology & Evolution 36, no. 4: 308–320. 33229137 10.1016/j.tree.2020.10.018

[ece370618-bib-0002] Allouche, O. , A. Tsoar , and R. Kadmon . 2006. “Assessing the Accuracy of Species Distribution Models: Prevalence, Kappa and the True Skill Statistic (TSS).” Journal of Applied Ecology 43, no. 6: 1223–1232.

[ece370618-bib-0003] Asamoah, E. F. , L. J. Beaumont , and J. M. Maina . 2021. “Climate and Land‐Use Changes Reduce the Benefits of Terrestrial Protected Areas.” Nature Climate Change 11, no. 12: 1105–1110.

[ece370618-bib-0004] Banks‐Leite, C. , R. M. Ewers , H. Folkard‐Tapp , and A. Fraser . 2020. “Countering the Effects of Habitat Loss, Fragmentation, and Degradation Through Habitat Restoration.” One Earth 3, no. 6: 672–676.

[ece370618-bib-0005] Benson, J. F. , P. J. Mahoney , J. A. Sikich , et al. 2016. “Interactions Between Demography, Genetics, and Landscape Connectivity Increase Extinction Probability for a Small Population of Large Carnivores in a Major Metropolitan Area.” Proceedings of the Royal Society B: Biological Sciences 283, no. 1837: 20160957. 10.1098/rspb.2016.0957 PMC5013791 27581877

[ece370618-bib-0006] Chen, G. , X. Li , and X. Liu . 2022. “Global Land Projection Based on Plant Functional Types With a 1‐km Resolution Under Socio‐Climatic Scenarios.” Scientific Data 9, no. 1: 1–18. 35354830 10.1038/s41597-022-01208-6 PMC8967933

[ece370618-bib-0007] Chen, R. , J. Carruthers‐Jones , S. Carver , and J. Wu . 2024. “Constructing Urban Ecological Corridors to Reflect Local Species Diversity and Conservation Objectives.” Science of the Total Environment 907, no. 10: 167987. 37875200 10.1016/j.scitotenv.2023.167987

[ece370618-bib-0008] Coetzee, B. W. T. , M. P. Robertson , B. F. N. Erasmus , B. J. Van Rensburg , and W. Thuiller . 2009. “Ensemble Models Predict Important Bird Areas in Southern Africa Will Become Less Effective for Conserving Endemic Birds Under Climate Change.” Global Ecology and Biogeography 18, no. 6: 701–710.

[ece370618-bib-0009] Colyn, R. B. , D. A. Ehlers Smith , Y. C. Ehlers Smith , H. Smit‐Robinson , and C. T. Downs . 2020. “Predicted Distributions of Avian Specialists: A Framework for Conservation of Endangered Forests Under Future Climates.” Diversity and Distributions 26, no. 6: 652–667.

[ece370618-bib-0010] Cushman, S. A. , B. Mcrae , F. Adriaensen , P. Beier , M. Shirley , and K. Zeller . 2013. “Biological Corridors and Connectivity.” Key Topics in Conservation Biology 2, no. 21: 384–404.

[ece370618-bib-0011] Dai, L. , Y. Liu , and X. Luo . 2021. “Integrating the MCR and DOI Models to Construct an Ecological Security Network for the Urban Agglomeration Around Poyang Lake, China.” Science of the Total Environment 754: 141868. 33254915 10.1016/j.scitotenv.2020.141868

[ece370618-bib-0012] Dirzo, R. , H. S. Young , M. Galetti , G. Ceballos , N. J. B. Isaac , and B. Collen . 2014. “Defaunation in the Anthropocene.” Science 345, no. 6195: 401–406. 25061202 10.1126/science.1251817

[ece370618-bib-0014] Elith, J. , H. Graham , J. Elith , et al. 2006. “Novel Methods Improve Prediction of Species' Distributions From Occurrence Data.” Ecography 29, no. 2: 129–151.

[ece370618-bib-0015] Elsen, P. R. , W. B. Monahan , E. R. Dougherty , and A. M. Merenlender . 2020. “Keeping Pace With Climate Change in Global Terrestrial Protected Areas.” Science Advances 6, no. 25: eaay0814. 32596440 10.1126/sciadv.aay0814 PMC7299617

[ece370618-bib-0016] Fagan, W. F. 2002. “Connectivity, Fragmentation, and Extinction Risk in Dendritic Metapopulations.” Ecology 83, no. 12: 3243–3249.

[ece370618-bib-0017] Feng, X. , C. Lin , H. Qiao , and L. Ji . 2015. “Assessment of Climatically Suitable Area for *Syrmaticus reevesii* Under Climate Change.” Endangered Species Research 28, no. 1: 19–31.

[ece370618-bib-0018] Gao, C. , H. Pan , M. Wang , et al. 2023. “Identifying Priority Areas for Ecological Conservation and Restoration Based on Circuit Theory and Dynamic Weighted Complex Network: A Case Study of the Sichuan Basin.” Ecological Indicators 155, no. 10: 111064.

[ece370618-bib-0019] Grainger, M. J. , P. J. Garson , S. J. Browne , P. J. K. McGowan , and T. Savini . 2018. “Conservation Status of *Phasianidae* in Southeast Asia.” Biological Conservation 220: 60–66.

[ece370618-bib-0020] Gurrutxaga, M. , and S. Saura . 2014. “Prioritizing Highway Defragmentation Locations for Restoring Landscape Connectivity.” Environmental Conservation 41, no. 2: 157–164.

[ece370618-bib-0021] Han, L. , Z. Wang , M. Wei , et al. 2022. “Small Patches Play a Critical Role in the Connectivity of the Western Tianshan Landscape, Xinjiang, China.” Ecological Indicators 144, no. 6: 109542.

[ece370618-bib-0022] Herrera, L. P. , M. C. Sabatino , F. R. Jaimes , and S. Saura . 2017. “Landscape Connectivity and the Role of Small Habitat Patches as Stepping Stones: An Assessment of the Grassland Biome in South America.” Biodiversity and Conservation 26, no. 14: 3465–3479.

[ece370618-bib-0024] Hijmans, R. J. , S. Phillips , J. Leathwick , and J. Elith . 2023. “Dismo: Species Distribution Modeling.” R Package Version 1, 3–14. https://CRAN.R‐project.org/package=dismo.

[ece370618-bib-0025] Hooper, D. U. , E. C. Adair , B. J. Cardinale , et al. 2012. “A Global Synthesis Reveals Biodiversity Loss as a Major Driver of Ecosystem Change.” Nature 486, no. 7401: 105–108. 22678289 10.1038/nature11118

[ece370618-bib-0026] Hu, L. , J. Long , Y. Lin , et al. 2022. “Arctic Introgression and Chromatin Regulation Facilitated Rapid Qinghai‐Tibet Plateau Colonization by an Avian Predator.” Nature Communications 13, no. 1: 6413. 10.1038/s41467-022-34138-3 PMC9613686 36302769

[ece370618-bib-0027] Huang, G. , X. Ping , W. Xu , et al. 2021. “Wildlife Conservation and Management in China: Achievements, Challenges and Perspectives.” National Science Review 8, no. 7: nwab042. 34691694 10.1093/nsr/nwab042 PMC8310758

[ece370618-bib-0028] IUCN . 2020. “The IUCN Red List of Threatened Species, e.T41643A17969392.” RLTS.T41643A17969392.en. 10.2305/IUCN.UK.2020-2.

[ece370618-bib-0029] Jones‐Farrand, D. T. , T. M. Fearer , W. E. Thogmartin , F. R. Thompson 3rd , M. D. Nelson , and J. M. Tirpak . 2011. “Comparison of Statistical and Theoretical Habitat Models for Conservation Planning: The Benefit of Ensemble Prediction.” Ecological Applications 21, no. 6: 2269–2282. 21939060 10.1890/10-1047.1

[ece370618-bib-0030] Keane, A. , M. D. L. Brooke , and P. J. K. McGowan . 2005. “Correlates of Extinction Risk and Hunting Pressure in Gamebirds (Galliformes).” Biological Conservation 126, no. 2: 216–233.

[ece370618-bib-0031] Kramer‐Schaadt, S. , E. Revilla , T. Wiegand , and U. Bretenmonser . 2004. “Fragmented Landscapes, Road Mortality and Patch Connectivity: Modelling Influences on the Dispersal of Eurasian *Lynx* .” Journal of Applied Ecology 41, no. 4: 711–723.

[ece370618-bib-0032] Le Roux, D. S. , K. Ikin , D. B. Lindenmayer , A. D. Manning , and P. Gibbons . 2018. “The Value of Scattered Trees for Wildlife: Contrasting Effects of Landscape Context and Tree Size.” Diversity and Distributions 24, no. 1: 69–81.

[ece370618-bib-0033] Li, D. , S. Wu , L. Liu , Y. Zhang , and S. Li . 2018. “Vulnerability of the Global Terrestrial Ecosystems to Climate Change.” Global Change Biology 24, no. 9: 4095–4106. 29804316 10.1111/gcb.14327

[ece370618-bib-0034] Li, W. B. , Y. Teng , M. Y. Zhang , et al. 2024. “Human Activity and Climate Change Accelerate the Extinction Risk to Non‐Human Primates in China.” Global Change Biology 30, no. 1: e17114. 38273577 10.1111/gcb.17114

[ece370618-bib-0035] Liu, Z. , Q. Huang , and G. Tang . 2021. “Identification of Urban Flight Corridors for Migratory Birds in the Coastal Regions of Shenzhen City Based on Three‐Dimensional Landscapes.” Landscape Ecology 36, no. 7: 2043–2057.

[ece370618-bib-0036] Liu, Z. , S. Tian , S. Lu , et al. 2023. “Climate and Land‐Use Changes Threaten the Effectiveness of Protected Areas for Protecting Galliformes in Southeast Asia.” Frontiers in Ecology and Evolution 11: 1216769.

[ece370618-bib-0037] Lu, Q. , P. Wang , J. Chang , et al. 2023. “Population Genomic Data Reveal Low Genetic Diversity, Divergence and Local Adaptation Among Threatened Reeves's Pheasant ( *Syrmaticus reevesii* ).” Avian Research 15: 100156.

[ece370618-bib-0038] McRae, B. H. , B. G. Dickson , T. H. Keitt , and V. B. Shah . 2008. “Using Circuit Theory to Model Connectivity in Ecology, Evolution, and Conservation.” Ecology 89, no. 10: 2712–2724. 18959309 10.1890/07-1861.1

[ece370618-bib-0039] McRae, B. H. , S. A. Hall , P. Beier , and D. M. Theobald . 2012. “Where to Restore Ecological Connectivity? Detecting Barriers and Quantify Restoration Benefits.” PLoS One 7, no. 12: e52604. 23300719 10.1371/journal.pone.0052604 PMC3531461

[ece370618-bib-0040] Mi, C. , L. Ma , M. Yang , et al. 2023. “Global Protected Areas as Refuges for Amphibians and Reptiles Under Climate Change.” Nature Communications 14, no. 1: 1389. 10.1038/s41467-023-36987-y PMC10011414 36914628

[ece370618-bib-0041] Minor, E. S. , and D. L. Urban . 2008. “A Graph‐Theory Framework for Evaluating Landscape Connectivity and Conservation Planning.” Conservation Biology 22, no. 2: 297–307. 18241238 10.1111/j.1523-1739.2007.00871.x

[ece370618-bib-0042] Murphy, S. J. , and A. B. Smith . 2021. “What Can Community Ecologists Learn From Species Distribution Models?” Ecosphere 12, no. 12: e03864.

[ece370618-bib-0043] National Forestry and Grassland Administration . 2021. “List of National Key Protected Wildlife.” http://www.yyj.moa.gov.cn/gzdt/202102/t20210205_6361296.htm.

[ece370618-bib-0044] Pascual‐Hortal, L. , and S. Saura . 2006. “Comparison and Development of New Graph‐Based Landscape Connectivity Indices: Towards the Priorization of Habitat Patches and Corridors for Conservation.” Landscape Ecology 21, no. 7: 959–967.

[ece370618-bib-0045] Pecchi, M. , M. Marchi , V. Burton , et al. 2019. “Species Distribution Modelling to Support Forest Management. A Literature Review.” Ecological Modelling 411: 108817.

[ece370618-bib-0046] Peng, J. , H. Zhao , and Y. Liu . 2017. “Urban Ecological Corridors Construction: A Review.” Shengtai Xuebao 37, no. 1: 23–30.

[ece370618-bib-0047] Peng, J. , S. Zhao , J. Dong , et al. 2019. “Applying Ant Colony Algorithm to Identify Ecological Security Patterns in Megacities.” Environmental Modelling and Software 117: 214–222.

[ece370618-bib-0048] Prevedello, J. A. , M. Almeida‐Gomes , and D. B. Lindenmayer . 2018. “The Importance of Scattered Trees for Biodiversity Conservation: A Global Meta‐Analysis.” Journal of Applied Ecology 55, no. 1: 205–214.

[ece370618-bib-0049] R Core Team . 2022. “R: A Language and Environment for Statistical Computing.” R Foundation for Statistical Computing. Vienna, Austria. https://www.R‐project.org/.

[ece370618-bib-0050] R Core Team . 2023. “R: A Language and Environment for Statistical Computing.” R Foundation for Statistical Computing. Vienna, Austria. https://www.R‐project.org/.

[ece370618-bib-0051] Rahbek, C. , and R. K. Colwell . 2011. “Species Loss Revisited.” Nature 473, no. 7347: 288–289. 21593855 10.1038/473288a

[ece370618-bib-0052] Razgour, O. , B. Forester , J. B. Taggart , et al. 2019. “Considering Adaptive Genetic Variation in Climate Change Vulnerability Assessment Reduces Species Range Loss Projections.” Proceedings of the National Academy of Sciences of the United States of America 116, no. 21: 10418–10423. 31061126 10.1073/pnas.1820663116 PMC6535011

[ece370618-bib-0053] Sannigrahi, S. , S. Bhatt , S. Rahmat , S. K. Paul , and S. Sen . 2018. “Estimating Global Ecosystem Service Values and Its Response to Land Surface Dynamics During 1995–2015.” Journal of Environmental Management 223: 115–131. 29908397 10.1016/j.jenvman.2018.05.091

[ece370618-bib-0054] Saura, S. , Ö. Bodin , and M. Fortin . 2014. “Stepping Stones Are Crucial for Species' Long‐Distance Dispersal and Range Expansion Through Habitat Networks.” Journal of Applied Ecology 51, no. 1: 171–182.

[ece370618-bib-0055] Saura, S. , and L. Pascual‐Hortal . 2007. “A New Habitat Availability Index to Integrate Connectivity in Landscape Conservation Planning: Comparison With Existing Indices and Application to a Case Study.” Landscape and Urban Planning 83, no. 2–3: 91–103.

[ece370618-bib-0056] Saura, S. , and J. Torné . 2009. “Conefor Sensinode 2.2: A Software Package for Quantifying the Importance of Habitat Patches for Landscape Connectivity.” Environmental Modelling & Software 24, no. 1: 135–139.

[ece370618-bib-0057] Tang, H. , J. Peng , H. Jiang , et al. 2023. “Spatial Analysis Enables Priority Selection in Conservation Practices for Landscapes That Need Ecological Security.” Journal of Environmental Management 345, no. 8: 118888. 37690245 10.1016/j.jenvman.2023.118888

[ece370618-bib-0071] Thompson, D. W. J., S. Solomon, P. J. Kushner, M. H. England, K. M. Grise, and D. J. Karoly. 2011. “Signatures of the Antarctic Ozone Hole In Southern Hemisphere Surface Climate Change.” Nature Geoscience 4, no. 11: 741–749.

[ece370618-bib-0058] Tian, S. , S. Lu , J. Hua , et al. 2022. “Integrating Habitat Suitability Modelling and Assessment of the Conservation Gaps of Nature Reserves for the Threatened Reeves's Pheasant.” Bird Conservation International 32: 384–389.

[ece370618-bib-0059] Tian, S. , J. Xu , and Y. Wang . 2020. “Human Infrastructure Development Drives Decline in Suitable Habitat for Reeves's Pheasant in the Dabie Mountains in the Last 20 Years.” Global Ecology and Conservation 22: e00940.

[ece370618-bib-0060] Ubachs, W. 2016. “A Testing Time for Antimatter.” Science 354, no. 6312: 546–547. 27811251 10.1126/science.aah6215

[ece370618-bib-0061] Visconti, P. , R. L. Pressey , D. B. Segan , and B. A. Wintle . 2010. “Conservation Planning With Dynamic Threats: The Role of Spatial Design and Priority Setting for Species' Persistence.” Biological Conservation 143, no. 3: 756–767.

[ece370618-bib-0062] Vogt, P. , and K. Riitters . 2017. “GuidosToolbox: Universal Digital Image Object Analysis.” European Journal of Remote Sensing 50, no. 1: 352–361.

[ece370618-bib-0063] Wan, X. , G. Jiang , C. Yan , et al. 2019. “Historical Records Reveal the Distinctive Associations of Human Disturbance and Extreme Climate Change With Local Extinction of Mammals.” Proceedings of the National Academy of Sciences 116, no. 38: 19001–19008. 10.1073/pnas.1818019116 PMC6754601 31481618

[ece370618-bib-0064] Xu, C. , Q. Yu , F. Wang , S. Qiu , M. Ai , and J. Zhao . 2023. “Identifying and Optimizing Ecological Spatial Patterns Based on the Bird Distribution in the Yellow River Basin, China.” Journal of Environmental Management 348, no. 2023: 119293. 37827082 10.1016/j.jenvman.2023.119293

[ece370618-bib-0065] Xu, J. L. , Z. W. Zhang , G. M. Zheng , X. H. Zhang , Q. H. Sun , and P. Mcgowan . 2007. “Home Range and Habitat Use of Reeves's Pheasant *Syrmaticus reevesii* in the Protected Areas Created From Forest Farms in the Dabie Mountains, Central China.” Bird Conservation International 17, no. 4: 319–330.

[ece370618-bib-0066] Zhang, K. , L. Yao , J. Meng , and J. Tao . 2018. “Maxent Modeling for Predicting the Potential Geographical Distribution of Two Peony Species Under Climate Change.” Science of the Total Environment 634: 1326–1334. 29710632 10.1016/j.scitotenv.2018.04.112

[ece370618-bib-0067] Zheng, G. 2015. “Pheasants in China.” Beijing, Higher Education Press. 1(4), 522–523.

[ece370618-bib-0068] Zheng, G. 2017. “A checklist on the Classification and Distribution of the Birds of China.” Beijing, Science Press. 3(5), 10.

[ece370618-bib-0069] Zhou, C. , J. Xu , and Z. Zhang . 2015. “Dramatic Decline of the Vulnerable Reeves's Pheasant *Syrmaticus reevesii* , Endemic to Central China.” Oryx 49, no. 3: 529–534.

[ece370618-bib-0070] Zhou, C. , Y. Zhao , J. W. Connelly , J. Li , and J. Xu . 2017. “Current Nature Reserve Management in China and Effective Conservation of Threatened Pheasant Species.” Wildlife Biology 7, no. 1: 1–9.

