# Supplementary material for: Land Use and Climate Change Accelerate the Loss of Habitat and Ecological Corridor to Reeves's Pheasant ( Syrmaticus reevesii ) in China
Source: Ecol Evol. 2024 Nov 26;14(11):e70618. doi: 10.1002/ece3.70618 (PMC11595527; doi:10.1002/ece3.70618)
Supplement: Supplementary file 1 — Table S1. Area of landscape type based on morphological spatial pattern analysis (MSPA) in 1995, 2020, and 2050. [file ECE3-14-e70618-s001.docx]

| Table S1 Area of landscape type based on Morphological Spatial Pattern Analysis MSPA in 1995, 2020 and 2050 | | | |
| --- | --- | --- | --- |
| Landscape type | Area in year (km²) | | |
|  | 1995 | 2020 | 2050 |
| Core | 72831.00 | 13239.80 | 8325.80 |
| Islet | 2619.36 | 198.40 | 183.20 |
| Loop | 3247.49 | 330.35 | 268.69 |
| Bridge | 3187.34 | 287.69 | 191.54 |
| Perforation | 3602.72 | 446.20 | 322.41 |
| Edge | 2703.75 | 524.85 | 412.92 |
| Branch | 3379.34 | 408.71 | 297.44 |
| Total | 91571.00 | 15436.00 | 10002.00 |
